# Simultaneous Determination of Salbutamol Sulphate and Bromhexine Hydrochloride in Tablets by Reverse Phase Liquid Chromatography

**DOI:** 10.4103/0250-474X.51957

**Published:** 2009

**Authors:** P. N. S Pai, G. K. Rao, M. S. Murthy, A. Agarwal, S. Puranik

**Affiliations:** Department of Quality Assurance, Al-Ameen College of Pharmacy, Bangalore-560 027, India

**Keywords:** Salbutamol sulphate, bromhexine hydrochloride, HPLC, UV detection

## Abstract

A simple reverse phase liquid chromatographic method has been developed and subsequently validated for simultaneous determination of salbutamol sulphate and bromhexine hydrochloride. The separation was carried out using a mobile phase consisting of acetonitrile, methanol and phosphate buffer, pH 4 in the ratio 60:20:20 v/v. The column used was SS Wakosil-II C-18 with a flow rate of 1 ml/min and UV detection at 224 nm. The described method was linear over a concentration range of 10-110 μg/ml and 20-140 μg/ml for the assay of salbutamol sulphate and bromhexine hydrochloride, respectively. The mean recovery was found to be 95-105% for salbutamol sulphate and 96.2-102.1% for bromhexine hydrochloride when determined at five different levels.

Salbutamol sulphate and bromhexine hydrochloride as components of a multi-ingredient formulation is useful in therapy of asthma. As found from the literature, salbutamol sulphate and bromhexine hydrochloride can be estimated individually by HPLC and spectrometric methods. Salbutamol sulphate and bromhexine hydrochloride has been reported to be estimated by spectrometric methods[1–8]. Salbutamol sulphate and bromhexine hydrochloride have been simultaneously determined by spectrometric[9] and HPLC methods[10]. The aim of the present work is to describe a liquid chromatographic procedure for the separation and simultaneous estimation of salbutamol sulphate and bromhexine hydrochloride in its formulation.

For the proposed method, acetonitrile HPLC grade, methanol HPLC grade, potassium dihydrogen phosphate, orthophosphoric acid and distilled water (Millipore) were used. The LC system consisted of Shimadzu LC-10AT pump, SS Wakosil-II C-18, 250×4.6 mm, 5 μm column, Rheodyne injector equipped with a 100 μl sample loop and UV Shimadzu SPD-10A VP detector, set at 224 nm. The output signal was monitored and integrated using Shimadzu CZ-RA software. Phosphate buffer pH 4, was prepared by dissolving 8.95 g of disodium hydrogen phosphate. 12 H_2_O and 3.40 g of potassium dihydrogen phosphate in 1000 ml of distilled water. The standard stock solution of salbutamol sulphate 1 mg/ml and bromhexine hydrochloride 1 mg/ml were prepared separately in mobile phase of acetonitrile, methanol and phosphate buffer, pH 4 in the ratio 60:20:20 v/v. The working standard solutions of salbutamol sulphate and bromhexine hydrochloride were prepared by diluting volumes ranging from 1 to 15 ml of standard stock solution of both the drugs separately to 100 ml with the mobile phase. The working standard solutions were injected into the chromatograph. The retention time for salbutamol sulphate and bromhexine hydrochloride at a flow rate of 1 ml/min were recorded as 4.2 and 6.3 min, respectively.

Analysis of marketed sample Mucolinc tablets (Cipla Ltd., Mumbai, India) of three different batches was carried out. Twenty tablets, each containing 2 mg salbutamol sulphate and 8 mg bromhexine hydrochloride were weighed. The tablets were crushed together in a mortar to a fine powder and an amount equivalent to 2 mg salbutamol sulphate and 8 mg bromhexine hydrochloride was transferred into a 100 ml dried volumetric flask. A few drops of acetonitrile were added to dissolve the active solids and then volume made up with the mobile phase to obtain sample stock solution. The solution was filtered through 0.45 μ Whatman filter paper. The sample solutions were injected into the stabilized liquid chromatographic system. From the respective peak areas obtained from the standard and sample chromatogram, the amount of salbutamol sulphate and bromhexine was calculated. The results of the analysis are tabulated in Table 1.

**TABLE 1:** DETERMINATION OF SALBUTAMOL SULPHATE AND BROMHEXINE HYDROCHLORIDE CONTENT IN MUCOLINC TABLETS

Batch	Content of salbutamol sulfate (mg/tablet)	Amount of salbutamol sulfate found* (mg/tablet)	Content of bromhexine hydrochloride (mg/tablet)	Amount of bromhexine hydrochloride found* (mg/tablet)
Bt-1	2	1.95	8	7.96
Bt-2	2	1.98	8	7.66
Bt-3	2	1.97	8	7.81

* Each value is an average of six determinations.

Accuracy of the method was checked by recovery studies, wherein sample was spiked with known quantity of standard drug of salbutamol sulphate and bromhexine hydrochloride at 5 different levels. The percentage recovery ranged from 95-105% for salbutamol sulphate and 96.2-102.1% for bromhexine hydrochloride. The precision of the method was studied by analysis of the mixture and expressed as percentage relative standard deviation, which was found to be 0.13% for salbutamol sulphate and 0.004% for bromhexine hydrochloride.

The linearity of the method was established by analysis of standard solution. The calibration curve was drawn by plotting the peak area versus concentration. The linearity range was found to be 10-110 μg/ml for salbutamol sulphate and 20-140 μg/ml for bromhexine hydrochloride. The specificity of the method was established by injecting placebo. No interference of the placebo was observed with the principal peaks. Ruggedness of the method was determined by carrying out the experiment on different instruments, by different chemists and on different days. The results showed that the method was rugged as percentage recovery was found to in the range of 95-100.5% for both of the drugs under study. The robustness of the method was determined by making slight changes in the chromatographic conditions. Buffer pH modification did not have any significant effect. The effect of organic strength on retention time was studied by small change in percentage polarity of the mobile phase system. It was found that even slight percentage change, up to 10% in ratio of mobile phase did not alter the position of the peaks.

The system suitability tests were carried out as per USP XXIV requirements. System suitability tests were carried out on freshly prepared standard stock solution of salbutamol sulphate and bromhexine and the parameters obtained with 100 μl injection volume. The number of theoretical plates for salbutamol sulphate and bromhexine hydrochloride was calculated as 19944 and 30337, respectively. The symmetry factor for salbutamol sulphate and bromhexine hydrochloride peak was found to be 1.08 and 1.11. The resolution between the two peaks was 1.85. The obtained results confirmed that the method is highly suitable for its intended purpose of separation of salbutamol sulphate and bromhexine hydrochloride and its simultaneous determination in tablet formulations.

In the reported method for estimation of salbutamol sulphate and bromhexine hydrochloride[10] the retention time has been reported to be more than 7 min. The proposed method is specific, accurate, rugged, robust, and precise as found from the laboratory studies. The method when applied for the determination of salbutamol sulphate and bromhexine hydrochloride in combined dosage marketed formulation, gave results conforming to the label claim of the drug.
